# The Frequency of “Brilliant” and “Genius” in Teaching Evaluations Predicts the Representation of Women and African Americans across Fields

**DOI:** 10.1371/journal.pone.0150194

**Published:** 2016-03-03

**Authors:** Daniel Storage, Zachary Horne, Andrei Cimpian, Sarah-Jane Leslie

**Affiliations:** 1 Department of Psychology, University of Illinois, Champaign, Illinois, United States of America; 2 Department of Philosophy, Princeton University, Princeton, New Jersey, United States of America; Northwestern University, UNITED STATES

## Abstract

Women and African Americans—groups targeted by negative stereotypes about their intellectual abilities—may be underrepresented in careers that prize brilliance and genius. A recent nationwide survey of academics provided initial support for this possibility. Fields whose practitioners believed that natural talent is crucial for success had fewer female and African American PhDs. The present study seeks to replicate this initial finding with a different, and arguably more naturalistic, measure of the extent to which brilliance and genius are prized within a field. Specifically, we measured field-by-field variability in the emphasis on these intellectual qualities by tallying—with the use of a recently released online tool—the frequency of the words “brilliant” and “genius” in over 14 million reviews on RateMyProfessors.com, a popular website where students can write anonymous evaluations of their instructors. This simple word count predicted both women’s and African Americans’ representation across the academic spectrum. That is, we found that fields in which the words “brilliant” and “genius” were used more frequently on RateMyProfessors.com also had fewer female and African American PhDs. Looking at an earlier stage in students’ educational careers, we found that brilliance-focused fields also had fewer women and African Americans obtaining bachelor’s degrees. These relationships held even when accounting for field-specific averages on standardized mathematics assessments, as well as several competing hypotheses concerning group differences in representation. The fact that this naturalistic measure of a field’s focus on brilliance predicted the magnitude of its gender and race gaps speaks to the tight link between ability beliefs and diversity.

## Introduction

Why are some fields more diverse than others? Although many factors are undoubtedly at play, a recent proposal suggests that the fields in which women and African Americans are underrepresented (e.g., physics, philosophy) are those fields whose members believe that a spark of brilliance is required for success [1]. The belief in the importance of untutored genius may be detrimental to the involvement of women and African Americans because of broad cultural stereotypes that portray the intellectual abilities of these groups in a negative light (e.g., [2, 3]). Consistent with this *Field-specific Ability Beliefs* (FAB) hypothesis, a recent survey of academics across 30 disciplines found that fields with a stronger focus on brilliance were also less diverse ([1, 4], see also [5]). In the present research, we examined this relationship using a different, and arguably more naturalistic, measure of the extent to which a field values brilliance and genius. Specifically, we tested whether differences between fields in the frequency of brilliance-related words (“brilliant” and “genius”) on RateMyProfessors.com, as captured by the recently released Gendered Language Tool [6], would track differences in the representation of women and African Americans. That is, fields in which students often comment on whether their professors are brilliant—a sign that this trait is prominent and valued—are expected to have larger gender and race gaps.

### Theoretical Background: The Field-specific Ability Beliefs Hypothesis

As mentioned above, the FAB hypothesis proposes that fields differ in the emphasis they place on raw intellectual talent and, further, that these differences affect the representation of groups that our culture portrays as lacking such talent [1]. This hypothesis builds on work examining the variability in individuals’ beliefs about success (e.g., [7, 8]). These beliefs fall along a continuum, with one end emphasizing the role of effort, strategies, and other such controllable factors (a growth mindset) and the other end focusing instead on raw, unchangeable talent as a source of success (a fixed mindset). One’s position on this continuum influences the goals and behaviors adopted in achievement settings. For instance, people with fixed (vs. growth) mindsets react more negatively to, and are generally warier of, mistakes because these could signal a lack of talent (for reviews, see [7–9]). By inducing a focus on looking effortlessly competent, fixed mindsets often prompt people to disengage from activities or contexts that might challenge them; as a result, fixed mindsets undermine persistence in domains or careers that are demanding (such as those in academia). The detrimental effects of fixed mindsets emerge particularly strongly in individuals with low confidence in their abilities, for whom the prospect of failure is more vivid and threatening (e.g., [10, 11]), but in the long term it seems no one is immune.

The mindsets one perceives in others are influential as well (e.g., [11, 12]). That is, people are attuned to the beliefs about success that prevail in a setting (e.g., classroom, department, workplace) and use these to inform their own goals and behaviors. The FAB framework extends this idea to the level of entire fields or careers. That is, it proposes that there is variability among fields of activity in terms of whether they are portrayed and perceived as requiring factors that are under one’s control (effort, strategies, etc.) or beyond one’s control (talent, giftedness, etc.). These field-specific ability beliefs (FABs) shape the practices and norms within each field, creating a distinctive atmosphere that orients aspiring members to the core values of its members. In terms of their effects on achievement behavior, these third-person, environmental mindsets are largely similar to first-person mindsets, with climates that prize brilliance promoting displays of competence and discouraging engagement with tasks that carry a risk of failure (e.g., [13])—much like individual-level fixed mindsets.

Importantly, however, brilliance-focused FABs may also give rise to systematic biases in the composition of a field. Environments that are geared toward identifying and grooming the next generation of intellectual superstars may systematically discourage members of social groups who, due to societal stereotypes, have—or expect others to have—less confidence in their intellectual abilities. Members of these groups may feel that they do not belong in such brilliance-focused fields (e.g., [12, 14]) and may experience greater feelings of anxiety and threat because of the (likely) prospect of being judged negatively through the lens of their group membership (e.g., [15, 16]). Although individual-level variability in mindsets exposes select individuals to these processes, field-level beliefs that emphasize sheer brainpower make entire groups vulnerable (specifically, groups that are stigmatized for their presumed lack of brilliance). As a result, the combination of FABs and cultural stereotypes may provide a particularly powerful means of understanding imbalances in the gender and race composition of fields across academia and industry. Of course, these are not the only factors that could lead to such imbalances. For instance, boys and girls receive different socialization about math and science, both in the classroom (e.g., [17]) and at home (e.g., [18–20]); African American children are more likely to attend high-poverty, low-performing schools (e.g., [21, 22]); and so on. While factors such as these are undoubtedly part of a complete explanation for gender and race gaps, our investigation here will focus specifically on field-specific ability beliefs as a predictor of the field-by-field pattern of women’s and African Americans’ (under)representation among bachelor’s and PhD degree holders.

Initial evidence supporting the FAB hypothesis was provided by a study of academics across 30 fields in science and engineering (STEM), the social sciences, and the humanities [1, 4]. To assess FABs, participants were asked to rate their agreement with several statements regarding what is required for success in their field. As predicted, fields that prized intellectual giftedness had significantly fewer women and African Americans earning PhDs. This relationship held over the entire sample of 30 fields, as well as when looking separately at STEM fields and at fields in the social sciences and the humanities. Moreover, this relationship held even when statistically adjusting for variables such as the work demands of these fields, their selectivity, and the GRE scores of their applicants.

### The Present Research

In the present research, we sought to provide a conceptual replication of the finding that women and African Americans are underrepresented in fields that emphasize intellectual giftedness. Rather than relying on survey methodologies, as in prior work [1, 5], here we measured a field’s emphasis on brilliance by analyzing the language used in course reviews on the popular website RateMyProfessors.com. In particular, we tallied the frequency with which college students taking courses in a particular field spontaneously commented on whether their professors were “brilliant” or a “genius.” Our assumption was that more frequent use of these terms within a field signals that students taking courses in that field routinely evaluate its members on their intellectual prowess, which might in turn suggest that the field as a whole values this trait. Thus, we hypothesized that this simple word count derived from students’ anonymous online evaluations can serve as a naturalistic proxy for a field’s emphasis on raw intellectual talent, which in prior work was assessed with survey questions about what is required for success [1, 5]. Moreover, we hypothesized that this linguistic measure of a field’s ability beliefs should also (inversely) predict whether women and African Americans pursue degrees in that field.

Minimal linguistic measures similar to ours have been used successfully in past research to examine psychological variables on a large scale. For instance, several studies have used word counts from the language used in online forums (e.g., blogs, chat rooms) to track changes in psychological climate following threatening events such as September 11 (e.g., [23, 24]). These word-count measures revealed the expected post-event increase in use of words indicating emotional negativity (e.g., “guilty”), cognitive processing (e.g., “think”), and orientation toward others and the community (e.g., “share”)—an increase that was followed by a gradual return to pre-event baselines [23]. Thus, simple word counts can be a powerful tool for studying macro-level psychological phenomena that are difficult to capture adequately otherwise. Here, we used them as a measure of the extent to which academic fields value brilliance and genius, expecting to find an inverse relationship between the frequency of brilliance-related words on RateMyProfessors.com and the diversity of a field. Beyond testing this key prediction, we used our word-count data to explore several other questions that are relevant to the FAB hypothesis (e.g., is there bias in attributions of brilliance?). We now go on to outline the four research questions we sought to answer with these data.

The background societal stereotypes that impugn the intelligence of groups such as women and African Americans are a core component of the FAB framework. There is already evidence for these stereotypes (e.g., [3, 25]), but our data allow us to document these stereotypes as well, at least with respect to gender. (Race information is not available for the instructors on RateMyProfessors.com.) We thus ask whether “brilliant” and “genius” are used more for male than for female instructors (Question #1). While we predict a gender bias in the attribution of intelligence-related superlatives, it shouldn’t be the case that *any* superlatives are used more often for male than for female instructors. We will thus also tally superlatives that speak more generally to instructors’ skill (such as “excellent” and “amazing”), expecting these to show a less gender-biased distribution.

Our main goal, which is to provide a conceptual replication of Leslie, Cimpian, Meyer, and Freeland’s [1] findings, is captured by Question #2: Does use of “brilliant” and “genius” on RateMyProfessors.com predict diversity at the PhD level? We expect to replicate these prior findings: Fields with more brilliance-related language on RateMyProfessors.com (which may indicate a more brilliance-oriented general climate) should have fewer female and African American PhDs. In contrast, the educational and career choices of groups who are not stereotyped as lacking brilliance, such as Asian Americans, should be unrelated to a field’s emphasis on brilliance.

Rather than simply looking at the raw relationship between climate and diversity, we will also compare the predictive power of our linguistic measure of a field’s climate against several alternative hypotheses concerning diversity in science and beyond. (Because the data for these alternative hypotheses are drawn primarily from Leslie, Cimpian, et al. [1], whose main focus was gender diversity, many of the alternatives concern women’s representation specifically.) One such hypothesis suggests that women place more value than men on relationships with their family, friends, and community, and are thereby less likely to pursue fields with workloads that interfere with these valued relationships (e.g., [26–28]). As a result, women may be underrepresented in fields that require long hours. Another competing hypothesis suggests that women are underrepresented in fields that privilege thinking systematically and abstractly (“systemizing”) over reasoning intuitively about mental states (“empathizing”) (e.g., [29, 30]). A third alternative possibility is that women and African Americans actually do lack some of the intellectual firepower of other groups (e.g., [31, 32], but see [33–36]) and are thus underrepresented in fields that are extremely selective (that is, fields that allow only the most capable to join their ranks). The fourth alternative is that women and African Americans are underrepresented in fields that rely heavily on mathematics, which may put these groups at a disadvantage [37, 38]. Contrary to these alternatives, we expect that use of “brilliant” and “genius” on RateMyProfessors.com will predict the field-by-field variability in PhD diversity above and beyond these other measures.

Finally, we also expect that the frequency of more-general superlatives (e.g., “excellent,” “amazing”) on RateMyProfessors.com won’t have as strong a relationship with diversity as the frequency of superlatives that pertain specifically to intellectual ability. Finding that use of these other superlatives does not track the underrepresentation of stigmatized groups would pinpoint more directly a field’s tendency to idolize brilliance as a potential influence on its diversity.

Although prior research on the FAB hypothesis has focused exclusively on diversity at the PhD level, this framework predicts that a field’s climate will be related to its diversity at other stages as well. Here, we extended the research on the FAB hypothesis to an earlier point in students’ educational careers. Specifically, we asked whether field-specific ability beliefs predict diversity at the bachelor’s level as well (Question #3). Because this is the first study to examine this question, we used both the language-based measure derived from RateMyProfessors.com and the survey-based measure collected by Leslie, Cimpian, et al. [1]. We predicted that fields that place a greater emphasis on brilliance would have fewer women and African Americans earning bachelor’s degrees.

Our final question (Question #4) concerns whether differences among fields in their endorsement of the “brilliance = males” stereotype (operationalized as differences in use of “brilliant” and “genius” for male vs. female instructors) contribute to the differences in women’s representation. (Again, because information about instructors’ race is not available on RateMyProfessors.com, this question focuses on gender specifically.) Although this negative stereotype is undoubtedly a key part of the FAB framework, the theory is in fact neutral as to whether field-by-field differences in its endorsement are centrally involved in the emergence of gender gaps. On the one hand, it seems reasonable to suppose that fields whose views of women are more negative will be less diverse. On the other hand, since the “brilliance = males” stereotype is part of the common ground shared by most members of our culture, its local levels in a discipline may be less important than women’s awareness that—due to this pervasive stereotype—their intellectual capacities could potentially be called into question. In this case, variation across fields in endorsement of this background stereotype may not be straightforwardly related to gender diversity. The RateMyProfessors.com data may allow us to differentiate between these alternatives, so we investigate whether fields with stronger stereotypes (measured as greater use of “brilliant” and “genius” for male vs. female instructors) are also less diverse.

## Methods

### Data on PhD Representation

The proportions of female, African American, and Asian American PhDs were obtained from the National Science Foundation’s (NSF) Survey of Earned Doctorates [39]. Note that the data publicly available from the NSF do not break down these statistics by gender and race simultaneously; only separate breakdowns by gender and by race are provided in the public-use data. As a result, we did not investigate the intersection of these dimensions in our analyses. For example, when we explored what predicts the representation of African Americans, we included both males and females in our analyses.

### Data on Bachelor’s Representation

The proportions of female, African American, and Asian American bachelor’s degrees were obtained from NSF’s Science and Engineering Indicators [40]. Because NSF does not report data on non-STEM disciplines at the bachelor’s level, our analysis of bachelor’s degrees was limited to STEM disciplines. Also, as with the Survey of Earned Doctorates [39], the public-use data on bachelor’s degrees are broken down by gender and race separately, not by the intersection of these dimensions.

### Brilliance Language Measure

**Fig 1:**
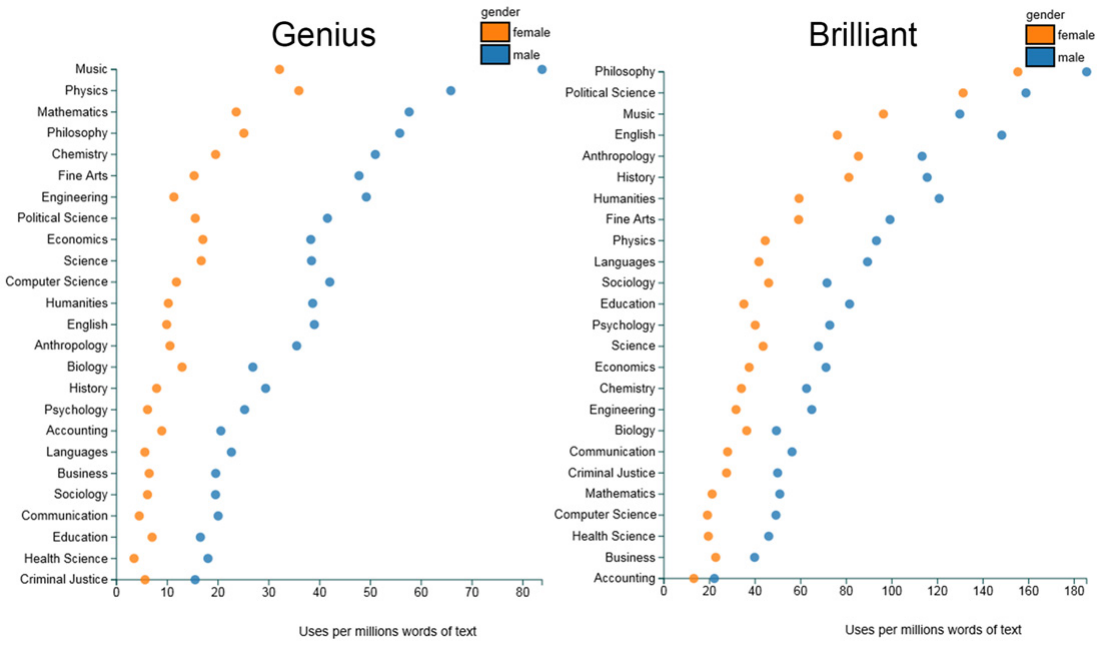
Frequency of “genius” and “brilliant” per millions of words of text on RateMyProfessors.com, split by gender and discipline.

The main independent variable—our new language-based measure of a field’s emphasis on raw intellectual talent—was calculated using the online Gendered Language Tool [6], which reports the number of uses of any given word per million words in RateMyProfessors.com reviews. More precisely, the tool reports a word’s frequency in each of 25 fields, separately for reviews of male and female instructors (see Fig 1). The tool searches over 14 million reviews from hundreds of different colleges and universities. The top three contributors to RateMyProfessors.com (and thus to the frequencies reported by the Gendered Language Tool) are the University of Central Florida, Miami Dade College, and San Diego State University. The data collected specifically for this study (namely, the word counts from the Gendered Language Tool) are completely anonymous and publicly available. Thus, the process of collecting them was exempt from review by an ethics committee.

We computed a *brilliance language score* for each discipline by (1) standardizing the frequencies of the words “brilliant” and (separately) “genius” for male and female instructors across the fields (which resulted in two *z*-scored variables, one for “brilliant” and one for “genius”), and then (2) averaging male and female instructors’ standardized scores for “brilliant” and “genius” within each field (4 scores) to derive a single number—the field’s brilliance language score.

The words “brilliant” and “genius” were chosen because they map most directly onto the intellectual traits that are prized in fields such as mathematics, physics, philosophy, etc. [1]. We found the same results, however, when we included the weaker term “smart” in the set of words denoting a brilliance focus. Thus, our results do not hinge on a particular configuration of search terms. It is also worth noting that other terms were considered but could not ultimately be used because they appeared very infrequently in the reviews (e.g., “gifted” was only used an average of 5.81 times per million words, vs. 75.10 for “brilliant” and 27.27 for “genius”) or because they do not uniquely target intellectual ability (e.g., a person can be “talented” in many ways).

We should point out that, because the brilliance language score is an average of male and female instructors’ separate averages, it weights the two gender-specific scores equally, and it is thus not influenced by whether there are more male or female instructors in a field. As a result, any relationships we identify between this score and women’s representation are not trivial—they are not simply the artifacts of correlating two different measures of gender diversity.

The same algorithm was used to construct the composite usage score for the control superlatives “excellent” and “amazing,” which were selected because they were roughly matched in intensity with the focal terms “brilliant” and “genius” (all being very positive) and were also used relatively frequently by students. However, similar results were found for analogous, but less frequent, control superlatives such as “fantastic” and “wonderful.” Thus, the results reported below are not specific to a particular set of control terms.

### Academics’ Ability Beliefs

The data on academics’ ability beliefs, as well as three of the four competing hypotheses (concerning a field’s workload, relative emphasis on systemizing vs. empathizing, and selectivity) were taken from Leslie, Cimpian, et al.’s study of academics [1]. We describe these measures briefly here and list the items in Table A in the S1 File. For full details, we refer the reader to [1] and its supplemental materials (http://bit.ly/1SP8k39). To assess field-specific ability beliefs, Leslie, Cimpian, et al. asked 1820 academics from 30 disciplines (both in and beyond STEM) to rate the extent to which they, as well as other people in their field, agree with four statements concerning what is required for success in their field (e.g., “Being a top scholar of [discipline] requires a special aptitude that just can’t be taught”). Participants’ ratings were averaged to create a composite measure of each field’s ability beliefs (α = 0.90).

### Competing Hypotheses

Leslie, Cimpian, et al. [1] assessed a field’s work demands by asking participants to report the number of hours they worked in a given week, both on and off campus (see Table A in the S1 File). To assess the extent to which a field relies on systemizing versus empathizing, Leslie, Cimpian, et al.’s participants were asked to rate the extent to which scholarly work in their discipline requires “identifying the abstract principles, structures, or rules that underlie the relevant subject matter” (systemizing; 2 items) or “recognizing and responding appropriately to people’s mental states” (empathizing; 2 items) (see Table A in S1 File). The average for the empathizing items (α = 0.90) was subtracted from the average for the systemizing items (α = .63) to create each field’s systemizing versus empathizing score. To assess the selectivity alternative, faculty members in Leslie, Cimpian, et al.’s [1] study were asked to estimate the proportion of graduate applicants who are accepted into their PhD program in any given year. Finally, to assess the extent to which a field relies on mathematics, we obtained field-level Quantitative GRE averages from the Educational Testing Service [41], on the assumption that math-intensive fields will have applicants with higher quantitative GRE scores.

## Results

Most of our analyses used 18 out of the 25 fields available in the Gendered Language Tool. The remaining 7 fields were not used either because they were too general (e.g., “science”) or because they could not be matched with the fields in Leslie, Cimpian, and colleagues’ [1] dataset (e.g., “criminal justice”; see Table B in S1 File for matching information, and Tables C and H in S1 File for the raw data). Below, we answer in turn each of the research questions discussed in the introduction.

### Question #1: Are “brilliant” and “genius” used more for male than for female instructors?

Across the 18 fields in our analysis, “brilliant” was used in a 1.81:1 male:female ratio and “genius” in a 3.10:1 ratio (see Fig 1). Both of these ratios were significantly different from a 1:1 ratio, one-sample *t*s(17) > 7.99, *p*s < .001, signaling a bias in favor of male instructors. In contrast, we found little evidence of gender bias in use of “excellent” and “amazing” in online evaluations, with male:female ratios of 1.08:1 and 0.91:1, respectively. Both of these ratios were significantly less male-skewed than the ratios for “brilliant” and “genius,” paired-sample *t*s(17) > 8.03, *p*s < .001. Thus, it is not the case that female instructors are viewed in an overall negative light. The female disadvantage seems specific to superlatives about intellectual ability, consistent with the existence of pervasive stereotypes against women on this dimension [3].

### Question #2: Does use of “brilliant” and “genius” on RateMyProfessors.com predict diversity at the PhD level?

In answering this question, we first examined the validity of the brilliance language score as a measure of field-specific ability beliefs. Does use of “brilliant” and “genius” on RateMyProfessors.com actually track a field’s focus on brilliance? We indeed found a tight link between the frequency of comments about brilliance and genius within a field and that field’s explicit emphasis on raw intellectual aptitude (as measured via a survey of academics in [1]), *r*(16) = .62 [.22, .85], *p* = .006. (Throughout, we present 95% confidence intervals in square brackets.) The more frequently the terms “brilliant” and “genius” were used on RateMyProfessors.com to evaluate instructors in a field, the more strongly academics in that field endorsed the importance of intellectual talent for success.

**Fig 2:**
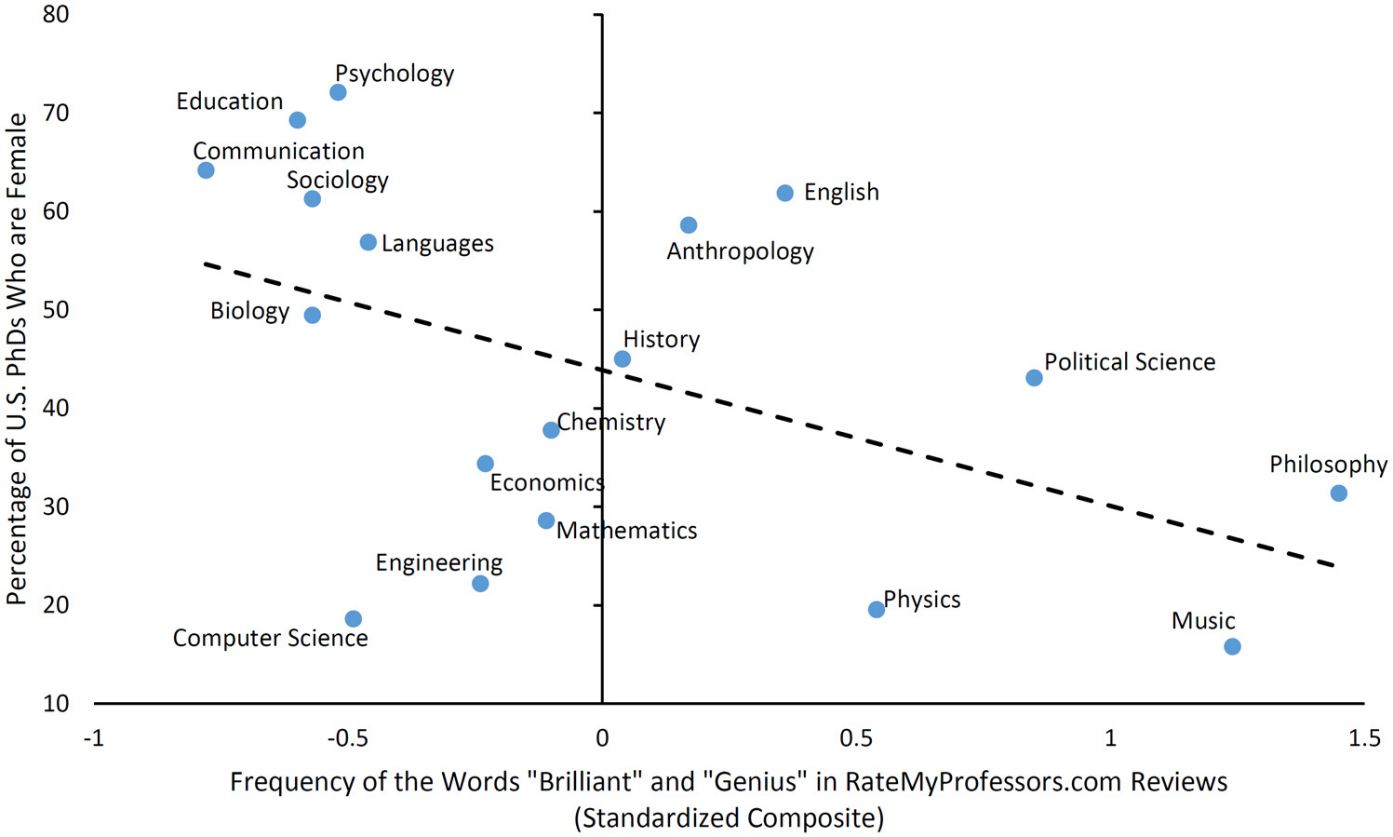
Use of the words “brilliant” and “genius” on RateMyProfessors.com predicts the percentage of 2011 U.S. PhDs who are female.

Next, we examined the relationship between the brilliance language score and the data on women’s representation at the PhD level. Replicating Leslie, Cimpian, and colleagues’ findings [1], we found that fields with more brilliance language on RateMyProfessors.com also had fewer female PhDs, *r*(16) = −.49 [−.78, −.02], *p* = .041 (see Fig 2). For comparison, the correlation between the survey-based FAB measure reported in [1] and female PhD representation over the 18 fields considered here was −.72 [−.89, −.39], *p* < 0.001. The difference between these two correlation coefficients was not statistically significant, *z* = −1.46, *p* = 0.145 [42, 43]. Nevertheless, the relationship between the brilliance language score and female PhD representation was reduced to zero when adjusting for the survey-based FAB score, *r*(15) = −.06 [−.53, .43], *p* = .807, whereas the relationship between the survey-based FAB score and female PhD representation was not affected to the same degree when partialling out the brilliance language score, *r*(15) = −.61 [−.84, −.19], *p* = .009. These analyses suggest that, unsurprisingly, Leslie, Cimpian, and colleagues’ data from academics [1] provide a more direct measure of a field’s emphasis on brilliance than the brilliance language score; as a result, the survey-based measure explains unique variance in PhD gender gaps (whereas the brilliance language score does not).

Importantly, the relationship between the brilliance language score and the gender diversity of a field cannot be explained by the greater use of “brilliant” and “genius” for male than female instructors (see Question #1). The imbalance in use of these adjectives would provide an alternative explanation for this relationship only if male instructors’ evaluations, which contain more brilliance language, were weighted more heavily in fields where there are more men, which was not the case. Moreover, the relationship between the brilliance language scores and women’s PhD attainment remained significant after adjusting for the four aforementioned competing hypotheses (namely, a field’s workload, relative emphasis on systematizing vs. empathizing, selectivity, and average Quantitative GRE score), as well as an indicator variable for whether a field is within STEM, *β* = −.48 [−.88, −.07], *p* = .025 (see Table 1). Although most of these controls are individually predictive of female representation [1, 4], they nonetheless failed to predict significant additional variance beyond our minimal measure of a field’s climate. Finally, note that brilliance language scores computed separately from male and female instructors’ evaluations were also predictive of gender gaps in PhD conferral above and beyond these four alternatives (see Table D in S1 File).

**Table 1:** Multiple regression analysis predicting female representation at the PhD level.

Predictor	*β*	*t*	*p*
STEM indicator variable	−.39	−1.27	.230
Brilliance language score	−.48*	−2.60	.025
Hours worked (on-campus)^a^	.26	0.98	.348
Systematizing vs. empathizing	.01	0.04	.971
Selectivity	.10	0.54	.597
Quantitative GRE	−.53	−1.62	.134
*R*^*2*^		77.9%	

* *p* < .05. *N* = 18 disciplines. “STEM” stands for “(Natural) Science, Technology, Engineering, and Mathematics.”

^a^ Although Leslie, Cimpian, et al. [1] collected data on the number of hours worked off campus as well, they found that the number of hours worked on campus was a better predictor of female representation than the total number of hours worked. Thus, to be conservative, we included this stronger competitor in our regression analyses. However, the brilliance language score remains a significant predictor even when the total number of hours worked (on- plus off-campus) is used in the regression.

**Fig 3:**
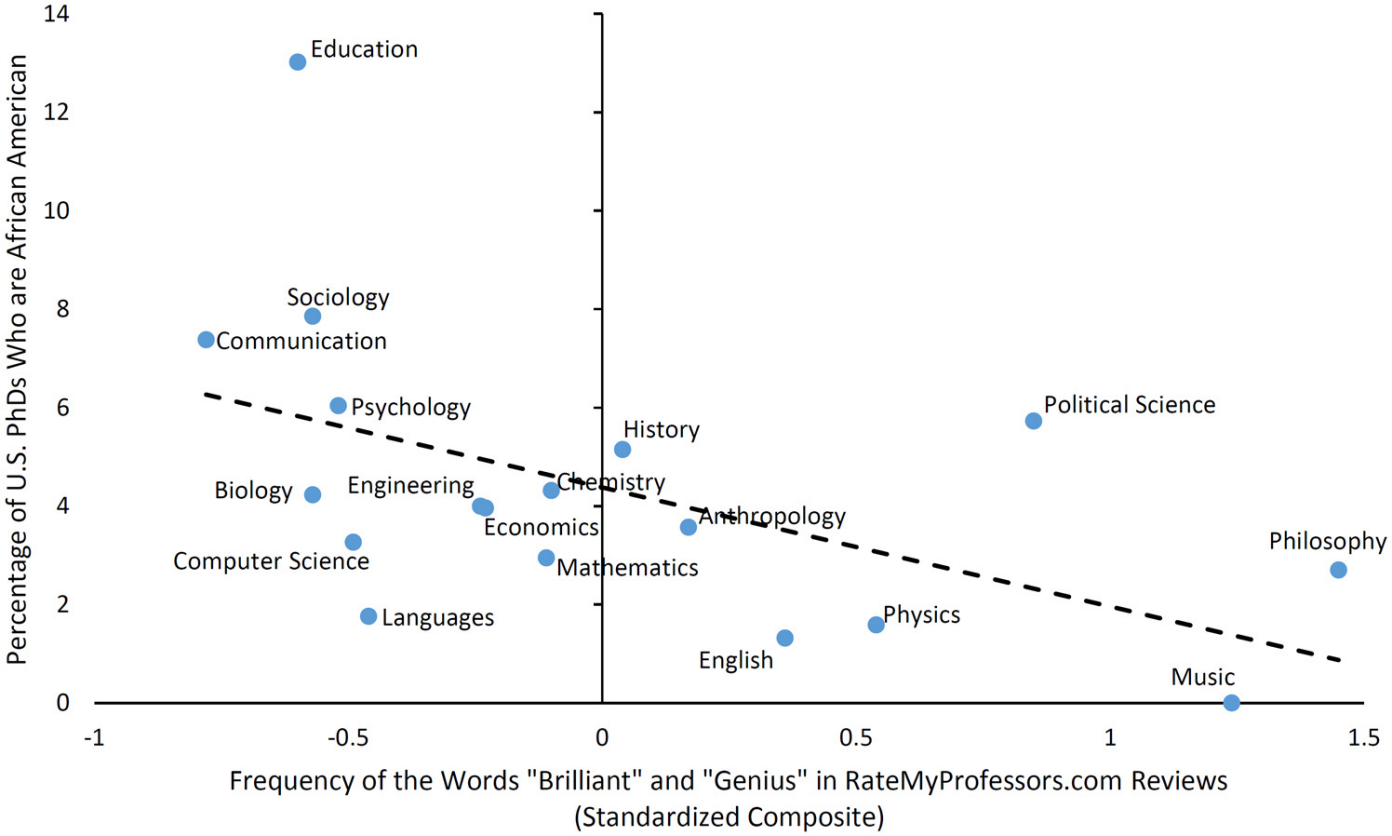
Use of the words “brilliant” and “genius” on RateMyProfessors.com predicts the proportion of 2011 U.S. PhDs who are African American.

Next, we tested whether the representation of African Americans at the PhD level might be similarly explained by the field-level variability in brilliance language scores. Consistent with our prediction—and again replicating Leslie, Cimpian, and colleagues’ findings [1]—fields in which “brilliant” and “genius” appeared more often on RateMyProfessors.com were also less likely to have African American PhDs, *r*(16) = −.53 [−.80, −.09], *p* = .023 (see Fig 3). For comparison, the correlation between the survey-based measure of FABs and African American PhD representation over these same 18 fields was −.73 [−.89, −.41], *p* < .001, which was not significantly different from the correlation with the brilliance language score, *z* = −1.29, *p* = .200. As before, however, partialling out each measure of FABs from the correlation of the other with African American PhD representation suggested that the survey-based FAB measure explains unique variance in race gaps (partial correlation for the survey-based measure adjusting for the brilliance language score: *r*[15] = −.61 [−.84, −.18], *p* = .010; partial correlation for the brilliance language score adjusting for the survey-based measure: *r*[15] = −.14 [−.58, .37], *p* = .596).

Notably, the brilliance language score remained a significant predictor of race gaps in PhD representation when adjusting for a field’s work demands, selectivity, and average Quantitative GRE scores, *β* = −.65 [−1.15, −0.14], *p* = .016 (see Table 2). None of these controls were themselves significant in the model. Regression models using the separate brilliance language scores computed from male and female instructors’ evaluations found these scores to also explain unique variance in African Americans’ PhD representation (see Table E in S1 File).

**Table 2:** Multiple regression analysis predicting African American representation at the PhD level.

Predictor	*β*	*t*	*p*
STEM indicator variable	−.32	–0.79	.447
Brilliance language score	−.65*	–2.80	.016
Hours worked (on-campus)	−.20	–0.53	.607
Selectivity	−.37	−1.40	.186
Quantitative GRE	−.09	–0.25	.806
*R*^*2*^		49.0%	

* *p* < .05. *N* = 18 disciplines. The brilliance language score was a significant predictor even in a model that included systemizing vs. empathizing (which was omitted from the main analysis above because it seemed uniquely relevant to the male vs. female contrast).

It is worth noting that the relationship between brilliance-related language on RateMyProfessors.com and African Americans’ PhD representation speaks against a possible alternative interpretation of the results concerning women’s representation: Perhaps fields that have more mentions of “brilliant” and “genius” in their online evaluations do so just because more undergraduate men take courses in them, and men may be more likely than women to value and comment on these traits (whereas women may be correspondingly more focused on the level of effort put in by their instructors; e.g., [44]). If so, the relationship between this language-based measure and women’s PhD attainment would simply amount to predicting fewer women at the PhD level based on observing fewer women in college. However, this alternative cannot explain why the frequency of “brilliant” and “genius” also predicts the representation of African Americans at the PhD level; no empirically documented differences in valuing brilliance vs. effort distinguish African Americans from other groups. Thus, the most parsimonious explanation for this set of findings is that our word-count measure indeed taps into a field’s shared beliefs about success. When these beliefs emphasize the need for brilliance, members of groups stereotypically viewed as lacking such a quality are less likely to obtain PhDs. Consistent with this interpretation, prior studies found that adjusting for the gender composition of the respondents from each discipline did not affect the predictive relationship between disciplines’ ability beliefs and their PhD diversity [1, 5]. Although such an adjustment is not possible here (since the gender of the students filling out evaluations on RateMyProfessors.com is not recorded), there is no reason to suppose that it would have any more of an effect on these relationships. With our current data, however, we cannot completely rule out this alternative.

To explore the divergent validity of our language-based measure of field climate, we tested whether the brilliance language score was a significant predictor of Asian Americans’ PhD attainment. We expected it might not be: The career aspirations of groups who are not targeted by negative stereotypes about intelligence shouldn’t be strongly affected by a field’s emphasis on brilliance. The results suggested that, although the relationship between the brilliance language score and the representation of Asian Americans at the PhD level was in the same direction as those for women and African Americans, it was of smaller magnitude and not significant, *r*(16) = –.25 [–.64, .24], *p* = .315. Brilliance language did not significantly predict Asian Americans’ Ph.D. representation beyond our controls either, *β* = −.22 [−.64, .20], *p* = .275 (see Table 3). This null result, combined with the significant results for women’s and African Americans’ PhD representation, supports the claim that groups who are the targets of negative stereotypes about their intelligence are particularly likely to be underrepresented in fields that cherish brilliance and genius.

**Table 3:** Multiple regression analysis predicting Asian American representation at the PhD level.

Predictor	*β*	*t*	*p*
STEM indicator variable	.31	0.91	.379
Brilliance language score	−.22	−1.14	.275
Hours worked (on-campus)	−.06	−0.20	.844
Selectivity	.15	0.66	.521
Quantitative GRE	.60~	2.06	.062
*R*^*2*^		65.1%	

^~^ *p* < .10. *N* = 18 disciplines.

The Gendered Language Tool allows word searches to be performed separately for positive vs. negative reviews (i.e., reviews that scored higher vs. lower than the midpoint of the “overall quality” rating on RateMyProfessors.com, respectively). In a separate set of analyses, we explored whether brilliance language scores computed separately over the positive and negative reviews predicted women’s and African Americans’ PhD representation. A priori, there is little reason to expect an asymmetry between these two language scores, since frequent use of “brilliant” and “genius” in reviews indicates a focus on intellectual ability regardless of whether these words are used to say something positive or negative about the instructor. (It is worth noting, however, that the most common reasons for negative reviews are probably unrelated to the instructor’s intelligence [e.g., “he’s a genius, but he can’t teach”].) As expected, the brilliance language scores derived from positive and negative reviews were significantly correlated with each other, *r*(16) = .51 [.06, .79], *p* = .029, and both were also correlated with women’s PhD representation (positive reviews: *r*(16) = −.45 [−.76, .02], *p* = .061; negative reviews: *r*(16) = −.65 [−.86, −.27], *p* = .003) and African Americans’ PhD representation (positive reviews: *r*(16) = −.49 [−.78, −.03], *p* = .039; negative reviews: *r*(16) = −.56 [−.81, −.12], *p* = .016). The separate brilliance language scores obtained from positive and negative reviews also predicted unique variance in PhD diversity above and beyond the relevant competing hypotheses (*β*s < −.50, *p*s < .024; see Tables F and G in S1 File). The only exception here was the regression predicting women’s representation based on the brilliance language from negative reviews, in which the coefficient for the brilliance language score was not significant, *β* = −.28 [−.89, .32], *p* = .322 (see Table F in S1 File). One possible reason for this result is that “brilliant” and “genius” were about three times less frequent in negative than in positive reviews; thus, the word tally based on the negative reviews was likely noisier.

Finally, we investigated the specificity of the link between the language used on RateMyProfessors.com and the underrepresentation of stigmatized groups: Does the frequency of other superlatives (beyond “brilliant” and “genius”) also predict gaps in PhD representation, or is this link specific to brilliance-related evaluative terms? Consistent with our argument, the frequency of the adjectives “excellent” and “amazing” was not significantly correlated with either women’s PhD representation, *r*(16) = .22 [−.27, .62], *p* = .378, or African Americans’ PhD representation, *r*(16) = .21 [−.29, .61], *p* = .413. This pattern of results suggests that it is the fields where people are judged on their *brilliance*—not just their skill—that have a problem attracting members of stigmatized groups.

### Question #3: Do field-specific ability beliefs predict diversity at the bachelor’s level as well?

Gender and race breakdowns for bachelor’s degrees were available for only 12 out of the 18 fields included in the preceding analyses [40] (see Table H in S1 File). Due to the considerably smaller sample, we calculated non-parametric rank-order correlations (Spearman’s ρ), which minimize the influence of extreme values (e.g., [45]). (Note, however, that the results were nearly identical with parametric [Pearson’s] correlations.) For the same reason, it was not feasible to adjust the correlation between ability beliefs and diversity in bachelor’s degrees for multiple control variables; instead, we partialled out just the average math SAT score of each field’s intended majors [46] (see Table H in S1 File), since the mathematical content of a field has been offered as an explicit alternative to FABs in the literature [47]. Finally, because the relationship between FABs and diversity at the bachelor’s level has not been investigated before, we conducted two sets of analyses: one with the survey-based FAB measure from Leslie, Cimpian, and colleagues’ study [1] and a second with the language-based FAB measure derived from RateMyProfessors.com.

Consistent with the FAB hypothesis, fields whose practitioners explicitly endorsed the value of intellectual talent (as indicated by the survey instrument) had fewer women obtaining bachelor’s degrees, ρ(10) = −.82 [−.95, −.46], *p* = .001, and African Americans, ρ(10) = −.63 [−.88, −.08], *p* = .029. Moreover, these correlations remained significant, or nearly so, when adjusting for math SAT scores (women: ρ[9] = −.77 [−.94, −.31], *p* = .006; African Americans: ρ[9] = −.54 [−.86, .09], *p* = .086). These partial correlations suggest that the relationship between a field’s emphasis on brilliance and the diversity of its bachelor’s degree holders is not explained by the extent to which the field relies on mathematics. In contrast, the partial correlations between math SAT scores and women’s and African Americans’ bachelor’s degrees adjusting for FAB scores were close to 0 (|ρ|s < .10, *p*s > .77). Finally, we found that Asian Americans were somewhat more likely to obtain bachelor’s degrees in disciplines that valued brilliance, ρ(10) = .56 [−.02, .86], *p* = .058 (partial correlation adjusting for math SAT: ρ[9] = .52 [−.12, .85], *p* = .104). To the extent that Asian Americans’ intellectual abilities may in fact be positively stereotyped (e.g., [48]), this result is broadly consistent with the FAB framework.

Next, we performed the same analyses using the brilliance language score. Although the relationships between the brilliance language score and the proportions of female and African American bachelor’s holders were in the predicted direction, the correlation coefficients were not statistically significant (women: ρ[10] = −.23 [−.71, .40], *p* = .471; African Americans: ρ[10] = −.41 [−.80, .21], *p* = .183). Given the indirect nature of the word-count measure and the small number of observations in this analysis, it is perhaps not surprising that the predicted relationships did not reach statistical significance. Similar to our analyses of the PhD data, the correlation between the brilliance language score and the distribution of bachelor’s degrees held by Asian Americans was smaller than those for women and African Americans, ρ(10) = −.06 [−.62, .53], *p* = .846.

Overall, the findings across these two sets of analyses suggest more support for the FAB hypothesis: When the brilliance focus of a field was measured directly by surveying academics, we found that women and African Americans (but not Asian Americans) are less likely to earn bachelor’s degrees in fields that cherish brilliance. The findings for the language-based FAB measure were weaker and should be interpreted with caution.

### Question #4: Are fields with stronger stereotypes (measured as greater use of “brilliant” and “genius” for male vs. female instructors) less diverse?

To explore this question, we first computed a field-specific male:female ratio of the uses of “brilliant” and “genius” to describe instructors on RateMyProfessors.com. Larger values of this ratio could be taken to indicate stronger stereotypes against women’s brilliance among the students taking courses in a field, which may also be reflective of broader attitudes within the field. We then tested whether these ratios were related to gender diversity at the PhD and bachelor’s levels. We focused more narrowly on gender diversity for this question because neither RateMyProfessors.com nor the Gendered Language Tool reports instructors’ race; thus, we were unable to compute the analogous stereotyping ratios for African Americans.

In terms of predictions, recall that the FAB hypothesis is compatible with multiple perspectives on this question. Although a negative relationship between a discipline’s level of stereotyping and its diversity would be consistent with the FAB framework, a weak or null relationship would be as well: The fact that the “brilliance = males” stereotype is at some level shared by most members of our cultural community (e.g., [3]) may be sufficient for its negative effects to emerge in fields that prize this intellectual trait; local, field-by-field variation in endorsement of this stereotype may be of only secondary importance. The results were more compatible with the latter possibility. That is, we found that the field-specific male:female ratios in the frequency of “brilliant” and “genius” were not significantly related to female representation either at the PhD level, *r*(16) = .20 [−.30, .61], *p* = .437, or at the bachelor’s level, ρ(10) = −.29 [−.74, .34], *p* = .354. However, caution is warranted in interpreting these null results, since the reliability and validity of the measure of stereotyping used in these analyses (the male:female word-count ratios) are far from certain.

## Discussion

A focus on brilliance in the comments posted on RateMyProfessors.com about instructors in a field consistently predicted lower involvement of women and African Americans—but not Asian Americans—in that field, even when taking into account other possible explanations for race and gender gaps in representation. These results provide a compelling conceptual replication of the earlier work that used explicit beliefs as a measure of a field’s brilliance focus [1, 4, 5].

Aside from providing a replication of these prior results, which would be a worthwhile goal in and of itself [49], the present study is valuable for several reasons. First, it relies on a wholly naturalistic measure of a field’s emphasis on brilliance. The college students whose reviews we used here were not filling out a questionnaire as part of a research study; rather, they were simply expressing their opinions about their instructors in an anonymous online forum. Yet, the frequency with which these students spontaneously commented on whether their instructors were “brilliant” and “geniuses” tracked not only academics’ own beliefs about the importance of these traits but also the magnitude of gender and race gaps across much of academia. Second, these naturalistic word-count data provide additional evidence for a “brilliance = males” stereotype. Across the fields represented on RateMyProfessors.com, superlatives about intelligence (but not ones about skill more generally) were used 2 to 3 times more often about male than about female instructors—a difference that further illustrates our culture’s negative attitudes toward women’s intellects. Third, the present research extends evidence for the FAB hypothesis to an earlier stage in students’ educational careers: bachelor’s degrees. Similar to the results on PhD diversity, we found that fields with stronger “cultures of genius” [13] had fewer female and African American (but not Asian American) students earning bachelor’s degrees. This new evidence suggests that field-specific ability beliefs begin to shape students’ career aspirations long before graduate school. In fact, given that these beliefs are, at least to a certain degree, endorsed by the general public as well (e.g., parents, teachers) [5], it is entirely possible that they influence youths before they even reach college.

### Limitations

The analyses reported here were limited in several ways. First, due to the structure of the Gendered Language Tool [6], word-count data were available only for a relatively small number of fields. Although the fields we examined are arguably among the largest (e.g., psychology, engineering), a wider range of fields would increase confidence in the generalizability of our conclusions. Second, because RateMyProfessors.com does not record the gender or race of the students leaving feedback, questions remain about the relationship between the demographic characteristics of the respondents and the frequency of brilliance-related words in their reviews (e.g., are males more likely to use “brilliant” and “genius”?). Third, the data available to us did not contain information on potential moderators of the relationship between brilliance language and diversity (e.g., the type of institution, the geographical region of the institution). Investigating such moderators must be left for future work. Despite these limitations, however, the present findings provide converging evidence for the relationship between field-specific ability beliefs and the involvement of women and African Americans across academia.

### Future Directions

We outline several questions that would be worthwhile to address in future work on this topic. First, more research is needed concerning the mechanisms responsible for the relationship between a field’s focus on raw intellectual ability and the underrepresentation of stigmatized groups. For instance, members of fields that cherish brilliance might be more likely to discriminate against students and colleagues from groups that are stereotypically seen as lacking such ability, offering them less support (e.g., [50, 51]) and fewer opportunities (e.g., [52, 53]). At the same time, the evaluative atmosphere in these fields might cause women and stigmatized minorities to worry that they will be judged on the basis of the stereotypes against their intelligence. This state of *stereotype threat* lowers the motivation and performance of those it affects (e.g., [16, 54]) and could thus lead women and African Americans to look for careers elsewhere.

Second, it would be worthwhile to explore how a field’s brilliance focus relates to its diversity at other career stages. The present study focused on the diversity of students at the bachelor’s and PhD levels, but would we see similar relationships with the diversity of, say, assistant professors, tenured professors, or endowed chairs? To speculate, given that women are likely to encounter additional, non-discipline-specific obstacles as their careers progress (e.g., inadequate childcare support; [55]), it is possible that the relationship between a field’s focus on brilliance and its gender diversity might attenuate with time.

Third, it is important to examine the developmental origins of the beliefs relevant to this phenomenon. When do children, for example, start believing that women’s intellectual abilities are inferior to men’s? What are the sources of this belief? Answers to these questions would be useful in part because they could inform interventions to encourage girls’ pursuit of “brilliance required” fields.

Another interesting, though perhaps less tractable, question concerns the reasons for the variability among fields in their beliefs about brilliance and genius. Why is it that some fields view these traits as essential for success and others do not? To what extent are these beliefs rooted in reality, and to what extent are they merely byproducts of a field’s history? Critically, however, even if these beliefs do track reality, they may nevertheless be discouraging for members of groups that are the targets of negative stereotypes about their intelligence.

## Conclusion

To conclude, the present study suggests that a focus on inherent intellectual abilities may discourage participation by groups who are stereotypically portrayed as lacking these abilities. In light of these data, it seems likely that turning the spotlight away from sheer brilliance—and toward the importance of sustained effort in achieving professional success [7, 8]—may bring about improvements in the diversity of many fields.

## Supporting Information

S1 File Supporting Information for *The Frequency of “Brilliant” and “Genius” in Teaching Evaluations Predicts the Representation of Women and African Americans across Fields* (PDF)
