# Supplementary material for: The Frequency of “Brilliant” and “Genius” in Teaching Evaluations Predicts the Representation of Women and African Americans across Fields
Source: PLoS One. 2016 Mar 3;11(3):e0150194. doi: 10.1371/journal.pone.0150194 (PMC4777431; doi:10.1371/journal.pone.0150194)
Supplement: S1 File — (PDF) [file pone.0150194.s001.pdf]

Supporting Information for

The Frequency of "Brilliant" and "Genius" in Teaching Evaluations Predicts the Representation of Women and African Americans across Fields

Table A

The measures from Leslie, Cimpian, et al.'s (2015) study that were used in the present research

---

Field-specific Ability Beliefs[a]

Being a top scholar of [discipline] requires a special aptitude that just can't be taught.
If you want to succeed in [discipline], hard work alone just won't cut it; you need to have an innate gift or talent.
With the right amount of effort and dedication, anyone can become a top scholar in [discipline]. (R)
When it comes to [discipline], the most important factors for success are motivation and sustained effort; raw ability is secondary. (R)

Hours Worked[b]

Approximately how many hours a week do you spend working:
- In your office, lab, classroom, or otherwise on campus?
- Off campus (e.g., home, coffee shop, other remote site)?

Systemizing vs. Empathizing[c]

Please rate the extent to which the following processes are involved in doing scholarly work in [discipline]:
- Identifying the abstract principles, structures, or rules that underlie the relevant subject matter (Systemizing)
- Analyzing the relevant subject matter and constructing a systematic understanding of it (Systemizing)
- Having a refined understanding of human thoughts and feelings (Empathizing)
- Recognizing and responding appropriately to people's mental states (Empathizing)

Selectivity[d]

Roughly what percentage of applicants are accepted into your department's PhD program in a typical year? (R)

Note. (R) indicates items that were reverse scored.
[a] Responses to these items were given on a 7-point scale (1 = strongly disagree to 7 = strongly agree).
[b] Responses to these items were given on an 8-point scale (1 to 8, 1-7 corresponding to 10-hour increments, and 8 corresponding to >70 hours).
[c] Response to these items were given on a 7-point scale (1 = never involved to 7 = highly involved).
[d] Responses to these items were given on a 10-point scale (1 to 10, each number corresponding to a 10% increment). There were two additional options for "don't know" and "no PhD program." This variable was reversed for analysis so that higher values indicate greater selectivity.

Table B

The fields matched between the Gendered Language Tool and Leslie, Cimpian, et al.'s (2015) dataset

Gendered Language Tool Fields	Leslie, Cimpian, et al. (2015) Fields
Accounting	N/A[a]
Anthropology	Archaeology[b], Anthropology
Biology	Biochemistry, Evolutionary Biology, Molecular Biology, Neuroscience
Business	N/A[a]
Chemistry	Chemistry
Communication	Communication
Computer Science	Computer Science
Criminal Justice	N/A[a]
Economics	Economics
Education	Education
Engineering	Engineering
English	Comparative Literature[b], English Literature
Fine Arts	N/A[a]
Health Science	N/A[a]
History	History
Humanities	N/A[a]
Languages	Classics[b], Linguistics, Spanish
Mathematics	Mathematics, Statistics
Music	Music Theory & Composition
Philosophy	Philosophy
Physics	Astronomy, Physics
Political Science	Political Science
Psychology	Psychology
Science	N/A[a]
Sociology	Sociology

Note. The matching was performed using the categories provided by the Educational Testing Service [41] as a guide. Weighted averages of different fields' values were computed where appropriate.

[a] "N/A" denotes that a field from the Gendered Language Tool was not matched with any of the fields from Leslie, Cimpian, et al.'s [1] dataset (*n* = 7).

[b] We performed a second set of analyses in which these fields were excluded, for a tighter match between the two datasets (e.g., some readers may disagree about whether Comparative Literature belongs under English). All significant results remain as reported in the main text.

Table C

The data on PhD diversity, quantitative GRE scores, survey-based FAB scores, competing hypotheses, and brilliance-related language

Field	STEM	% fem. PhDs	% Afr. Am. PhDs	Quant GRE	FAB	Hrs.	S vs. E	Selec -tivity	"Brlnt" and "genius" comp.	"Exlnt" and "amzg" comp.	"Brlnt" M	"Brlnt" F	"Ge-nius" M	"Ge-nius" F	"Exlnt" M	"Exlnt" F	"Amzg" M	"Amzg" F
Anthropology	0	58.60	3.57	149	3.73	3.35	1.33	1.73	0.17	-0.37	113.26	85.33	35.47	10.53	243.49	258.75	421.11	439.11
Biology	1	49.48	4.22	154	3.96	5.13	3.30	2.68	-0.57	-0.23	49.36	36.33	26.84	12.91	312.48	301.86	319.58	364.07
Chemistry	1	37.80	4.32	158	4.11	5.73	3.82	4.00	-0.10	-0.59	62.57	33.99	50.96	19.51	283.42	277.61	296.18	329.55
Comm.	0	64.20	7.38	149	3.79	3.38	1.26	1.84	-0.78	-0.34	56.20	27.95	20.01	4.48	268.22	257.70	375.04	451.24
Comp. Sci.	1	18.60	3.27	157	4.29	3.84	3.15	1.64	-0.49	-0.12	49.15	19.11	41.99	11.80	434.86	353.21	172.25	186.86
Economics	0	34.40	3.96	160	4.37	4.09	2.83	2.18	-0.23	-1.12	71.10	37.41	38.25	17.00	276.35	249.94	201.39	231.24
Education	0	69.30	13.02	149	3.32	3.12	1.01	3.20	-0.60	1.49	81.44	35.08	16.52	7.02	465.77	355.85	525.19	564.52
Engineering	1	22.20	4.00	159	4.29	4.55	3.38	3.38	-0.24	0.19	64.85	31.64	49.18	11.30	462.24	375.14	202.07	207.91
English	0	61.87	1.32	149	4.36	2.79	1.27	2.01	0.36	-0.02	148.20	76.10	38.95	9.88	301.47	277.90	421.85	443.75
History	0	45.00	5.15	148	3.90	2.87	1.16	2.24	0.04	-0.02	115.49	81.12	29.36	7.90	328.99	282.76	404.68	392.70
Languages	0	56.89	1.76	150	4.11	3.45	2.26	1.77	-0.46	1.08	89.32	41.67	22.62	5.59	395.22	367.01	479.82	534.12
Mathematics	1	28.60	2.95	162	4.57	3.72	4.53	2.59	-0.11	-0.15	50.84	21.16	57.62	23.57	338.03	322.51	267.81	355.52
Music	0	15.80	0.00	150	4.45	3.22	2.18	3.40	1.24	1.16	129.80	96.29	83.77	32.10	313.75	338.23	589.58	704.44
Philosophy	0	31.40	2.70	153	5.11	2.71	3.01	1.29	1.45	0.01	185.45	155.28	55.76	25.08	293.31	298.84	407.59	444.14
Physics	1	19.56	1.59	161	4.33	4.68	3.98	3.27	0.54	-0.88	93.23	44.45	65.82	35.90	284.05	256.46	265.89	259.88
Political Sci.	0	43.10	5.73	151	3.94	3.60	2.56	2.18	0.85	-0.14	158.82	131.26	41.53	15.51	315.26	271.31	382.86	406.73
Psychology	0	72.10	6.04	149	3.55	3.79	1.43	1.59	-0.52	0.35	72.75	40.06	25.22	6.11	312.10	303.19	459.25	513.04
Sociology	0	61.30	7.86	149	3.78	3.33	2.37	2.38	-0.57	-0.28	71.54	45.95	19.50	6.11	260.36	261.71	383.27	480.68

Note. FAB = academics' field-specific ability beliefs. Hrs. = hours worked (on campus). S vs. E = systemizing vs. empathizing score. "Brlnt" = "brilliant." "Exlnt" = "excellent." "Amzg" = "amazing." The values for FAB, Hrs., S vs. E, and Selectivity were all taken from Leslie, Cimpian, et al.'s [1] dataset. The composite scores were calculated by (1) standardizing the frequencies of the two relevant terms (separately) across all fields, and then (2) averaging male and female instructors' standardized scores for the two relevant terms within each field. The PhD representation data is for the year 2011.

Table D

*Multiple regression analysis predicting **female representation** at the PhD level based on separate word counts for the male and the female instructors*

	**Male** instructors' evaluations			**Female** instructors' evaluations		
Predictor	β	t	p	β	t	p
STEM indicator variable	−.34	−1.15	0.276	−.43	−1.31	0.217
**Brilliance language score**	**−.48***	**−2.69**	**0.021**	**−.45***	**−2.32**	**0.040**
Hours worked (on-campus)	.21	0.80	0.441	.33	1.27	0.229
Systematizing vs. empathizing	−.05	−0.14	0.894	.05	0.13	0.900
Selectivity	.07	0.38	0.712	.15	0.80	0.438
Quantitative GRE	−.47	−1.46	0.172	−.60	−1.72	0.114
R^2		78.5%			76.1%	

* p < .05.

Table E

Multiple regression analysis predicting ***African American representation*** *at the PhD level based on separate word counts for the male and the female instructors*

	**Male** instructors' evaluations			**Female** instructors' evaluations		
Predictor	*β*	*t*	*p*	*β*	*t*	*p*
STEM indicator variable	−.29	−0.80	0.440	−.33	−0.72	0.487
**Brilliance language score**	**−.75****	**−3.46**	**0.005**	**−.51~**	**−2.05**	**0.063**
Hours worked (on-campus)	−.32	−0.91	0.378	−.05	−0.12	0.906
Selectivity	−.45~	−1.82	0.094	−.28	−0.98	0.347
Quantitative GRE	−.02	−0.07	0.949	−.18	−0.47	0.645
R^2		57.7%			37.4%	

~ $p < .10$. * $p < .05$. ** $p < .01$.

Table F

*Multiple regression analysis predicting **female representation** at the PhD level based on separate word counts for positive and negative reviews*

	**Positive** evaluations			**Negative** evaluations		
Predictor	*β*	*t*	*p*	*β*	*t*	*p*
STEM indicator variable	−.38	−1.32	0.213	−.29	−0.75	0.468
**Brilliance language score**	**−.50***	**−2.95**	**0.013**	**−.28**	**−1.04**	**0.322**
Hours worked (on-campus)	.23	0.94	0.366	.51	1.76	0.106
Systematizing vs. empathizing	<.01	0.01	0.990	−.26	−0.55	0.593
Selectivity	.11	0.62	0.546	.16	0.69	0.504
Quantitative GRE	−.55	−1.77	0.104	−.34	−0.84	0.419
R^2		80.1%			67.5%	

* $p < .05$.

Table G

Multiple regression analysis predicting ***African American representation*** *at the PhD level based on separate word counts for positive and negative reviews*

	**Positive** evaluations			**Negative** evaluations		
Predictor	*β*	*t*	*p*	*β*	*t*	*p*
STEM indicator variable	−.30	−0.71	0.490	−.42	−0.99	0.343
**Brilliance language score**	**−.62***	**−2.62**	**0.022**	**−.77***	**−2.61**	**0.023**
Hours worked (on-campus)	−.19	−0.51	0.622	.14	0.40	0.699
Selectivity	−.35	−1.28	0.225	−.42	−1.50	0.158
Quantitative GRE	−.15	−0.42	0.679	.24	0.56	0.584
R^2		46.4%			46.1%	

* $p < .05$.

Table H

The data on bachelor’s diversity and mathematics SAT scores (Question #3)

Fields	% female bachelor’s	% African American bachelor’s	% Asian American bachelor’s	Math SAT
Anthropology	70.09	4.94	6.81	553
Biological Sciences	59.57	7.13	15.71	552
Chemistry	49.10	7.12	13.94	582
Computer Science	17.67	10.14	8.12	547
Economics	30.61	4.71	14.96	553
Engineering	18.77	3.97	11.24	580
Linguistics	68.31	3.32	10.84	539
Mathematics & Statistics	42.98	4.56	10.22	604
Physics & Astronomy	20.36	3.84	9.16	582
Political Science	51.51	9.78	6.44	553
Psychology	76.96	11.46	6.31	490
Sociology	69.65	17.80	6.44	553

Note. The bachelor’s degree data are for the year 2011 and were taken from Appendix Tables 2-17 (*Earned bachelor’s degrees, by sex and field: 2000–11*) and 2-23 (*Earned bachelor’s degrees, by citizenship, field, and race or ethnicity: 2000–11*) in NSF’s *Science and Engineering Indicators* [40]. The SAT scores were taken from Table 25 (*Intended College Major, Degree-Level Goal*) in The College Board’s 2013 *College-Bound Seniors: Total Group Profile Report* [46].
